# Supplementary material for: From sequence to enzyme mechanism using multi-label machine learning
Source: BMC Bioinformatics. 2014 May 19;15:150. doi: 10.1186/1471-2105-15-150 (PMC4229970; doi:10.1186/1471-2105-15-150)
Supplement: Additional file 2 — Java code of ml2db. Additional file ml2db_code.tar.gz contains the Java source code to run the multi-label machine learning experiments and save the results to database. The code’s Javadoc is included. [file 1471-2105-15-150-S2.zip › additional file 2/ml2db/utils/doc/index-files/index-19.html]

S-Index


JavaScript is disabled on your browser.


- Overview
- Package
- Class
- Use
- Tree
- Deprecated
- Index
- Help

- Prev Letter
- Next Letter

- Frames
- No Frames

- All Classes

A B C D E F G H I J K L M N O P Q R S T U V W X Y 


## S

saveRow(TreeMap<String, String>) - Method in class uk.ac.standrews.utils.main.database.Table


Scripts - Class in uk.ac.standrews.utils.main
:   Class

Scripts() - Constructor for class uk.ac.standrews.utils.main.Scripts


SECOND - Static variable in class uk.ac.standrews.utils.main.TimeUtils


separatedString(Vector<String>, String) - Static method in class uk.ac.standrews.utils.main.collectionutils.CollectionUtils


Set - Class in uk.ac.standrews.utils.main.collectionutils.setutils
:   A set and its subsets.

Set(String, TreeSet<String>, SupersetsManager) - Constructor for class uk.ac.standrews.utils.main.collectionutils.setutils.Set


Set(String, TreeSet<String>, SupersetsManager, TreeSet<Set>) - Constructor for class uk.ac.standrews.utils.main.collectionutils.setutils.Set


Set - Class in uk.ac.standrews.utils.main.setutils
:   A set and its subsets.

Set(String, TreeSet<String>, SupersetsManager) - Constructor for class uk.ac.standrews.utils.main.setutils.Set


Set(String, TreeSet<String>, SupersetsManager, TreeSet<Set>) - Constructor for class uk.ac.standrews.utils.main.setutils.Set


setAdded(int) - Method in class uk.ac.standrews.utils.main.diff.Difference
:   Sets the point as added.

setAttribute(String, String) - Method in class uk.ac.standrews.utils.main.webutils.simpledomparser.XmlNode


setChosenOption(String) - Method in class uk.ac.standrews.utils.main.guiutils.SimpleRadioButtonPanel


setColumns2Values(TreeMap<String, String>) - Method in class uk.ac.standrews.utils.main.database.TableRow


setCookie(String, String) - Method in class uk.ac.standrews.utils.main.webutils.ClientHttpRequestUtils
:   adds a cookie to the requst

setCookies(Map) - Method in class uk.ac.standrews.utils.main.webutils.ClientHttpRequestUtils
:   adds cookies to the request

setCookies(String[]) - Method in class uk.ac.standrews.utils.main.webutils.ClientHttpRequestUtils
:   adds cookies to the request

setDeleted(int) - Method in class uk.ac.standrews.utils.main.diff.Difference
:   Sets the point as deleted.

setDiag(boolean) - Method in class edu.cornell.lassp.houle.RngPack.Ranlux
:   Turns diagnostic messages on and off.

setFromList(ArrayList<String>) - Static method in class uk.ac.standrews.utils.main.collectionutils.ListUtils
:   create a set (elements appear only once) from a list

setFromObjectsToString(TreeSet<Object>) - Static method in class uk.ac.standrews.utils.main.Utils


setInitialisation(boolean) - Method in interface uk.ac.standrews.utils.main.Initialisable


setInitialisation(boolean) - Method in class uk.ac.standrews.utils.main.Initialised


setInitialisationIsCorrect() - Method in class uk.ac.standrews.utils.main.Initialised


setInitialisationNotCorrect() - Method in class uk.ac.standrews.utils.main.Initialised


setIsPrimaryKey(boolean) - Method in class uk.ac.standrews.utils.main.database.TableColumn
:   Sets the column primary key status

setManager(Manager) - Method in class uk.ac.standrews.utils.main.database.Managed


setParameter(String, File) - Method in class uk.ac.standrews.utils.main.webutils.ClientHttpRequestUtils
:   adds a file parameter to the request

setParameter(String, Object) - Method in class uk.ac.standrews.utils.main.webutils.ClientHttpRequestUtils
:   adds a parameter to the request; if the parameter is a File, the file is
    uploaded, otherwise the string value of the parameter is passed in the
    request

setParameter(String, String) - Method in class uk.ac.standrews.utils.main.webutils.ClientHttpRequestUtils
:   adds a string parameter to the request

setParameter(String, String, InputStream) - Method in class uk.ac.standrews.utils.main.webutils.ClientHttpRequestUtils
:   adds a file parameter to the request

setParameters(Map) - Method in class uk.ac.standrews.utils.main.webutils.ClientHttpRequestUtils
:   adds parameters to the request

setParameters(Object[]) - Method in class uk.ac.standrews.utils.main.webutils.ClientHttpRequestUtils
:   adds parameters to the request

setState(double, double) - Method in class cern.jet.random.Pareto
:   Sets the parameters.

setTagName(String) - Method in class uk.ac.standrews.utils.main.webutils.simpledomparser.XmlNode


SetTest - Class in uk.ac.standrews.utils.test.setutils
:   Class

SetTest() - Constructor for class uk.ac.standrews.utils.test.setutils.SetTest


setText(String) - Method in class uk.ac.standrews.utils.main.webutils.simpledomparser.XmlNode


setToString(TreeSet<String>) - Static method in class uk.ac.standrews.utils.main.setutils.SetUtils


setUp() - Method in class uk.ac.standrews.utils.test.database.DbConnPropsTest
:   the database connection properties file for test (database = test)

setUp() - Method in class uk.ac.standrews.utils.test.database.DbCreatorTest


setUp() - Method in class uk.ac.standrews.utils.test.database.DbManagerTest


setUp() - Method in class uk.ac.standrews.utils.test.database.DbReaderTest


setUp() - Method in class uk.ac.standrews.utils.test.database.DbWriterTest


setUp() - Method in class uk.ac.standrews.utils.test.database.SqlUtilsTest


setUp() - Method in class uk.ac.standrews.utils.test.database.TableCreatorTest


setUp() - Method in class uk.ac.standrews.utils.test.database.TableReaderTest


setUp() - Method in class uk.ac.standrews.utils.test.database.TableRowTest


setUp() - Method in class uk.ac.standrews.utils.test.database.TableTest


setUp() - Method in class uk.ac.standrews.utils.test.database.TableWriterTest


setUp() - Method in class uk.ac.standrews.utils.test.FileUtilsTest


setUp() - Method in class uk.ac.standrews.utils.test.NumberUtilsTest


setUp() - Method in class uk.ac.standrews.utils.test.SimpleDOMParserTest


setUp() - Method in class uk.ac.standrews.utils.test.WebUtilsTest


setUp() - Method in class uk.ac.standrews.utils.test.XmlNodeTest


setUp() - Method in class uk.ac.standrews.utils.test.XmlSearcherTest


setUp() - Method in class uk.ac.standrews.utils.test.XmlUtilsTest


SetUtils - Class in uk.ac.standrews.utils.main.collectionutils.setutils
:   Class

SetUtils() - Constructor for class uk.ac.standrews.utils.main.collectionutils.setutils.SetUtils


SetUtils - Class in uk.ac.standrews.utils.main.setutils
:   Class

SetUtils() - Constructor for class uk.ac.standrews.utils.main.setutils.SetUtils


similarElementExistsInTable(String, String, String, DbConn) - Static method in class uk.ac.standrews.utils.main.database.DbUtils


simpleDirectoryChooser() - Static method in class uk.ac.standrews.utils.main.guiutils.GuiUtils
:   Returns the directory chosen by the user in a file chooser window.

SimpleDOMParser - Class in uk.ac.standrews.utils.main.webutils.simpledomparser
:   `SimpleDOMParser` is a highly-simplified XML DOM parser.

SimpleDOMParser() - Constructor for class uk.ac.standrews.utils.main.webutils.simpledomparser.SimpleDOMParser


SimpleDOMParserTest - Class in uk.ac.standrews.utils.test
:   Class

SimpleDOMParserTest() - Constructor for class uk.ac.standrews.utils.test.SimpleDOMParserTest


simpleFileChooser() - Static method in class uk.ac.standrews.utils.main.guiutils.GuiUtils
:   Returns the file chosen by the user in a file chooser window.

simpleInputDialog(String, String) - Static method in class uk.ac.standrews.utils.main.guiutils.GuiUtils
:   Creates a simple input pop-up

simpleMessagePopUp(String) - Static method in class uk.ac.standrews.utils.main.guiutils.GuiUtils
:   Pop-up window with message (icon: information > "i")

SimpleRadioButtonPanel - Class in uk.ac.standrews.utils.main.guiutils


SimpleRadioButtonPanel(String[], String[]) - Constructor for class uk.ac.standrews.utils.main.guiutils.SimpleRadioButtonPanel
:   Generates a simple radio button popup

SINGLE\_QUOTE\_CHARACTER - Static variable in class uk.ac.standrews.utils.main.database.DbUtils


splitTextInLines(String) - Static method in class uk.ac.standrews.utils.main.RegExpUtils
:   I am splitting the String based on a regular expression which looks for
    carriage return (\r) or line feed (\n) or carriage return immediately
    followed by line feed.

SqlUtils - Class in uk.ac.standrews.utils.main.database
:   Class

SqlUtils() - Constructor for class uk.ac.standrews.utils.main.database.SqlUtils


SqlUtilsTest - Class in uk.ac.standrews.utils.test.database
:   Class

SqlUtilsTest() - Constructor for class uk.ac.standrews.utils.test.database.SqlUtilsTest


START\_TIMESTAMP - Static variable in class uk.ac.standrews.utils.main.database.DbUtils


staticNextDouble(double, double) - Static method in class cern.jet.random.Pareto
:   Returns a random number from the distribution with the given scale = k,
    and shape = alpha.

StatUtils - Class in uk.ac.standrews.utils.main.stats
:   Utilities to print simple histograms, percent of frequency etc

StatUtils() - Constructor for class uk.ac.standrews.utils.main.stats.StatUtils


StatUtilsTest - Class in uk.ac.standrews.utils.test.stats
:   Class

StatUtilsTest() - Constructor for class uk.ac.standrews.utils.test.stats.StatUtilsTest


STRING - Static variable in class uk.ac.standrews.utils.main.database.SqlUtils
:   Basic data type: string (anything that is not an integer or a double...)

string\_is\_empty(String) - Static method in class uk.ac.standrews.utils.main.stringutils.StringUtils


stringExistsInFile(String, String) - Static method in class uk.ac.standrews.utils.main.FileUtils


stringIsNullOrEmpty(String) - Static method in class uk.ac.standrews.utils.main.stringutils.StringUtils


stringMatchesRegExp(String, String, boolean) - Static method in class uk.ac.standrews.utils.main.RegExpUtils
:   matches a string against a regular expression (gives true if there is at
    least a partial match)

StringPool - Class in uk.ac.standrews.utils.main.stringutils
:   Class

StringPool(int) - Constructor for class uk.ac.standrews.utils.main.stringutils.StringPool


stringToArrayOfArrays(String, String) - Static method in class uk.ac.standrews.utils.main.collectionutils.ArrayUtils
:   Reads a special-character-separated string (eg comma or tab separated)
    and creates an array of rows-columns.

StringUtils - Class in uk.ac.standrews.utils.main.stringutils
:   Class

StringUtils() - Constructor for class uk.ac.standrews.utils.main.stringutils.StringUtils


StringUtilsTest - Class in uk.ac.standrews.utils.test
:   Class

StringUtilsTest() - Constructor for class uk.ac.standrews.utils.test.StringUtilsTest


stripFirstLastCharacter(String) - Static method in class uk.ac.standrews.utils.main.stringutils.StringUtils


stripXmlHtmlTags(String) - Static method in class uk.ac.standrews.utils.main.RegExpUtils
:   Strips all tags from xml or html code

substituteMatchedString(String, String, String) - Static method in class uk.ac.standrews.utils.main.RegExpUtils
:   Substitute an old string with a new string if the old string matches the
    given regular expression

suite() - Static method in class uk.ac.standrews.utils.test.AllDatabaseUtilsTests


suite() - Static method in class uk.ac.standrews.utils.test.AllMapUtilsTests


suite() - Static method in class uk.ac.standrews.utils.test.AllUtilsTests


suite() - Static method in class uk.ac.standrews.utils.test.stats.AllStatsUtilsTests


sumArray(double[]) - Static method in class uk.ac.standrews.utils.main.collectionutils.ArrayUtils


SupersetsManager - Class in uk.ac.standrews.utils.main.collectionutils.setutils
:   A class to identify the unique supersets among sets of interpro signatures.

SupersetsManager(String, DbManager, String) - Constructor for class uk.ac.standrews.utils.main.collectionutils.setutils.SupersetsManager


SupersetsManager(TreeMap<String, String>, String) - Constructor for class uk.ac.standrews.utils.main.collectionutils.setutils.SupersetsManager


SupersetsManager - Class in uk.ac.standrews.utils.main.setutils
:   A class to identify the unique supersets among sets of interpro signatures.

SupersetsManager(String, DbManager, String) - Constructor for class uk.ac.standrews.utils.main.setutils.SupersetsManager


SupersetsManager(TreeMap<String, String>, String) - Constructor for class uk.ac.standrews.utils.main.setutils.SupersetsManager


SupersetsManagerTest - Class in uk.ac.standrews.utils.test.setutils
:   Class

SupersetsManagerTest() - Constructor for class uk.ac.standrews.utils.test.setutils.SupersetsManagerTest


surroundWith(String, String) - Static method in class uk.ac.standrews.utils.main.database.SqlUtils
:   Adds a surrounding string (usually an apex or similar) as prefix and
    suffix of the given text.

SystemUtils - Class in uk.ac.standrews.utils.main
:   Utilities for the System class.

SystemUtils() - Constructor for class uk.ac.standrews.utils.main.SystemUtils

A B C D E F G H I J K L M N O P Q R S T U V W X Y

- Overview
- Package
- Class
- Use
- Tree
- Deprecated
- Index
- Help

- Prev Letter
- Next Letter

- Frames
- No Frames

- All Classes
